# Eosinophils, malignant brain edema, and functional outcome after mechanical thrombectomy: a mediation analysis

**DOI:** 10.3389/fneur.2026.1939369

**Published:** 2026-10-07

**Authors:** Haojun Lu, Zhiliang Guo, Shuhong Yu, Jinping Yang, Guodong Xiao

**Affiliations:** 1 Department of Neurology, Zhangjiagang Hospital of Traditional Chinese Medicine, Suzhou, China; 2 Department of Neurology, The Second Affiliated Hospital of Soochow University, Suzhou, China; 3 Department of Encephalopathy, Suzhou Hospital of Integrated Traditional Chinese and Western Medicine, Suzhou, China; 4 Department of Encephalopathy, Taicang Hospital of Nanjing University of Chinese Medicine, Suzhou, China

**Keywords:** eosinophils, malignant brain edema (MBE), mechanical thrombectomy, outcome, stroke

## Abstract

**Objective:**

To examine the independent associations of admission eosinophil count with malignant brain edema (MBE) and with poor 3-month functional outcome after mechanical thrombectomy (MT), and to quantify the statistical contribution of MBE to the eosinophil-outcome association using mediation analysis.

**Methods:**

A total of 442 consecutive AIS patients who underwent MT between May 2017 and January 2023 were analyzed. Multivariable logistic regression analysis was used to assess the association of eosinophils with MBE and poor outcome. Mediation analysis was performed to quantify the statistical contribution of MBE.

**Results:**

MBE developed in 98 (22.17%) patients. After full adjustment for confounders, each 1-standard deviation increase in standardized eosinophils was independently associated with a lower risk of MBE (OR = 0.165; 95% CI: 0.055–0.498; *p* = 0.00137). Consistent findings were observed when eosinophils were dichotomized by the median value (OR = 0.168; 95% CI: 0.086–0.328; *p* < 0.00001). Standardized eosinophils were also independently associated with reduced risk of poor outcome (OR = 0.620; 95% CI: 0.475–0.810; *p* = 0.00045). Mediation analysis showed a small statistical contribution of MBE to the eosinophil-outcome association: the indirect effect was −0.2109 (95% CI: −0.4016 to −0.0500; *p* < 0.001), and the mediated proportion was 12.05% (95% CI: 2.92–26%).

**Conclusion:**

Lower admission eosinophil levels were independently associated with higher risks of MBE and poor 3-month functional outcome. Mediation analysis indicated that MBE statistically accounted for 12.05% (95% CI: 2.92–26%) of the eosinophil-outcome association. These findings are hypothesis-generating and require external multicenter validation.

## Introduction

Mechanical thrombectomy (MT) is now the standard treatment for acute ischemic stroke (AIS) caused by large vessel occlusion (1, 2). However, malignant brain edema (MBE) can lead to deterioration of neurological function, thereby mitigating the clinical benefits of MT. The potential mechanism of MBE occurrence is closely linked to blood–brain barrier (BBB) disruption caused by ischemia/reperfusion (I/R) injury after MT (3–6)

Eosinophils secrete a variety of chemokines and vascular endothelial growth factor (VEGF) (7–9). Chemokines secreted by eosinophils induce the activation of M2 phenotype microglia, which exert neuroprotective effects and facilitate the resolution of inflammation. VEGF may protect neural tissue by regulating angiogenesis (7–9). These preclinical observations raise the possibility that reduced eosinophil counts may correlate with aggravated cerebral I/R injury and BBB breakdown. However, eosinophils do not exert exclusively protective effects: under distinct pathological contexts, they may also release pro-inflammatory mediators that exacerbate vascular damage, and their net role in post-stroke pathophysiology remains incompletely understood. Building on these unresolved questions, we hypothesized that MBE may act as a statistical intermediate linking admission eosinophil levels to 3-month functional outcome, rather than a confirmed causal mediator. This study aimed to examine the independent associations of eosinophils with MBE and functional outcome, and to quantify the statistical contribution of MBE to the eosinophil-outcome association using mediation analysis.

## Methods

### Study population

Consecutive acute ischemic stroke (AIS) patients who received endovascular treatment at the Second Affiliated Hospital of Soochow University were prospectively enrolled in the Suzhou Stroke Registry between May 2017 and January 2023. We conducted a retrospective analysis for this study. The eligibility criteria were: (1) age ≥ 18 years; (2) time from stroke onset to arterial puncture ≤24 h; (3) baseline National Institutes of Health Stroke Scale (NIHSS) score ≥6; and (4) receipt of MT. The exclusion criteria were as follows: (1) patients treated with intra-arterial thrombolysis only (*n* = 107); (2) patients with posterior circulation infarction (*n* = 73); (3) patients with incomplete peripheral blood cell counts, asthma, eosinophilic esophagitis, eosinophilic hypercellular syndrome, signs of active infection, chronic inflammatory diseases, autoimmune diseases, malignancy, severe liver or kidney dysfunction, or hematological disorders (*n* = 13); and (4) patients with unavailability of post-MT head CT imaging, incomplete clinical data, or loss to follow-up (*n* = 24). After excluding a total of 217 ineligible patients, 442 patients with AIS were finally included in the analysis (Participant Selection Flowchart: Appendix Figure I). The study adhered to the Helsinki Declaration. This retrospective study used de-identified clinical data, so informed consent was waived. The study protocol was approved by the Ethics Committee of the Second Affiliated Hospital of Soochow University (JD-HG-2025-078).

### Clinical protocol and laboratory tests

Baseline characteristics included age, sex, atrial fibrillation, hypertension, diabetes mellitus, hyperlipidemia, history of stroke, coronary artery disease, smoking and alcohol consumption, stroke etiology, National Institutes of Health Stroke Scale (NIHSS) score, Alberta Stroke Program Early CT Score (ASPECTS), and intravenous thrombolysis (IVT) history. Onset-to-puncture time (OPT) was also recorded. Stroke etiological subtypes were classified according to the Trial of Org 10,172 in Acute Stroke Treatment (TOAST) criteria, including atherosclerotic, cardioembolic, and other subtypes (rare-cause and unexplained causes) (2, 10). Peripheral venous blood samples were collected immediately upon hospital admission as part of routine emergency workup. The exposure of interest was the admission eosinophil count. All patients were followed up via telephone or outpatient visits for 3 months. A modified Rankin Scale (mRS) score greater than 2 at 3 months after admission was defined as poor outcome (2, 10).

### Assessment of MBE

All patients underwent cranial CT on admission. Follow-up CT scans were performed immediately after mechanical thrombectomy (MT) and again within 24–48 h postoperatively. Emergency CT re-examination was arranged if patients experienced acute neurological deterioration. According to previously published criteria, MBE was defined as a midline shift greater than 5 mm at the septum pellucidum or pineal gland on follow-up CT acquired within 5 days after MT (11, 12).

### Statistical analysis

Continuous variables with non-normal distribution were expressed as median (interquartile range, IQR) and compared using the Mann–Whitney *U* test; normally distributed continuous variables were presented as mean ± standard deviation and analyzed with the independent-samples *t-*test. Categorical variables were reported as counts (percentages) and compared using the chi-square test or Fisher’s exact test.

Multivariable logistic regression models were used to assess the associations of eosinophils with MBE and poor outcome. Eosinophils were incorporated into models as a continuous variable and a categorical variable dichotomized by the median value (0.01 × 10^9^/L). Raw eosinophil counts had a narrow numerical range, and a 1-unit increase (×10^9^/L) was clinically implausible, which would lead to unstable regression results. To address this issue, we applied *Z*-score standardization with the formula: *Z* = (X – 0.026) / 0.049. The mean and standard deviation of raw eosinophil counts in this cohort were 0.026 and 0.049, respectively. After transformation, the standardized variable had a mean and standard deviation close to the theoretical values of 0 and 1.

Covariate screening was performed as detailed in Appendix Tables II–V (2, 10, 13). Model 1 was adjusted for age and sex. Model 2 was further adjusted for variables associated with study outcomes at *p* < 0.10, or variables that altered the effect estimate of eosinophils by more than 10%. All subgroup analyses were exploratory, and no adjustment for multiple testing was applied. Likelihood ratio tests were used to evaluate interaction effects of eosinophils across subgroups.

Mediation analysis was performed to examine the statistical relationship among eosinophils, MBE, and outcome (14). All mediation analyses are statistical and exploratory in nature, and are not intended to establish biological causal pathways. The term “mediator” is used herein in its statistical sense only. The temporal sequence in this study—baseline eosinophil measurement at admission, MBE assessment on follow-up CT within 5 days post-MT, and 3-month functional outcome evaluation—satisfies the temporal order assumption for mediation analysis. However, temporal order alone does not establish causation. We calculated the total effect, direct effect, and indirect effect of eosinophils on 3-month outcome mediated by MBE. All statistical analyses were performed using EmpowerStats^1^ (X&Y Solutions, Boston, MA) and R software (version 4.5.1). A two-tailed *p* < 0.05 was defined as statistically significant.

## Results

### Study cohort

A total of 442 AIS patients (median age: 68 years) who underwent MT were included in the analysis. MBE was present in 98 (22.17%) patients and absent in 344 (77.83%). Baseline characteristics stratified by MBE status are summarized in Table 1. Patients with MBE had significantly higher baseline NIHSS scores (18.5 vs. 16.0; *p* < 0.001), a higher number of thrombectomy passes (2 vs. 1; *p* < 0.001), more extensive ischemic lesions as indicated by lower ASPECTS (7.00 [6.00–7.00] vs. 7.00 [7.00–8.00]; *p* = 0.002), and a higher proportion of female patients (54.08% vs. 40.99%; *p* = 0.021) and atrial fibrillation (57.14% vs. 42.15%; *p* = 0.009). The admission eosinophil count was significantly lower in the MBE group (median 0.00 × 10^9^/L [IQR 0.00–0.01] vs. 0.01 × 10^9^/L [IQR 0.00–0.04]; *p* < 0.001).

**Table 1:** Baseline characteristics of study participants according to the presence/absence of MBE.

Characteristics	All patients	No MBE	MBE	*p-*value
No. of patients	442	344	98	
Age, median (IQR), y	68.00 (58.00–75.00)	68.00 (57.00–76.00)	67.00 (60.00–75.00)	0.833
Female, *n* (%)	194 (43.89%)	141 (40.99%)	53 (54.08%)	0.021
Hypertension, *n* (%)	300 (67.87%)	230 (66.86%)	70 (71.43%)	0.393
Diabetes, *n* (%)	86 (19.46%)	66 (19.19%)	20 (20.41%)	0.787
Hyperlipidemia, *n* (%)	128 (28.96%)	104 (30.23%)	24 (24.49%)	0.269
Atrial fibrillation, *n* (%)	201 (45.48%)	145 (42.15%)	56 (57.14%)	0.009
Coronary heart disease, *n* (%)	72 (16.29%)	57 (16.57%)	15 (15.31%)	0.765
History of stroke, *n* (%)	64 (14.48%)	46 (13.37%)	18 (18.37%)	0.215
Smoking, *n* (%)	139 (31.45%)	109 (31.69%)	30 (30.61%)	0.840
Drinking, *n* (%)	103 (23.30%)	84 (24.42%)	19 (19.39%)	0.299
Baseline NIHSS, median (IQR)	17.00 (13.00–20.00)	16.00 (13.00–19.00)	18.50 (16.00–22.00)	<0.001
ASPECTS, median (IQR)	7.00 (7.00–8.00)	7.00 (7.00–8.00)	7.00 (6.00–7.00)	0.002
IVT, *n* (%)	139 (31.45%)	115 (33.43%)	24 (24.49%)	0.093
Stroke etiology, *n* (%)				0.061
LAA	192 (43.44%)	156 (45.35%)	36 (36.73%)	
Cardioembolic	223 (50.45%)	164 (47.67%)	59 (60.20%)	
Others	27 (6.11%)	24 (6.98%)	3 (3.06%)	
OPT, median (IQR), min	285 (220.00–380.00)	285.00 (211.50–394.00)	294.50 (232.00–350.00)	0.792
Number of passes, median (IQR)	2.00 (1.00–3.00)	1.00 (1.00–2.00)	2.00 (2.00–3.00)	<0.001
mTICI≥2b	399 (90.27%)	315 (91.84%)	84 (85.71%)	0.069
Eosinophils, median (IQR), 10^9^/l	0.01 (0.00–0.03)	0.01 (0.00–0.04)	0.00 (0.00–0.01)	<0.001
mRS, median (IQR)	3.00 (1.00–4.00)	3.00 (1.00–4.00)	6.00 (4.00–6.00)	<0.001
Poor outcome, n (%)	278 (62.90%)	191 (55.52%)	87 (88.78%)	<0.001

ASPECTS, Alberta Stroke Program Early CT Score; IQR, interquartile range; IVT, intravenous thrombolysis; LAA, large-artery atherosclerosis; mRS, modified Rankin Scale; mTICI, modified Thrombolysis in Cerebral Infarction; NIHSS, National Institutes of Health Stroke Scale; OPT, onset-to-puncture time.

### Relationships between eosinophils and MBE

Multivariable logistic regression results are summarized in Table 2. After multivariable adjustment (Model 2), the standardized eosinophil count (per 1-SD increase) was independently associated with a lower risk of MBE (OR = 0.165; 95% CI: 0.055–0.498; *p* = 0.00137). For sensitivity analysis, eosinophils were dichotomized by the median value (0.01 × 10^9^/L), and consistent inverse associations were observed (OR = 0.168; 95% CI: 0.086–0.328; *p* < 0.00001). Threshold effect analysis suggested that the *p* value for the log-likelihood ratio test was 1.000, indicating that a standard linear model (without a threshold) adequately fitted the relationship between eosinophils and MBE (Appendix Table VI).

**Table 2:** Relationship between eosinophils and MBE and functional outcome among patients with acute ischemic stroke in different models.

Variable	Non-adjusted model		Model 1		Model 2	
	*β*/OR (95%CI)	*P-*value	*β*/OR (95%CI)	*P-*value	*β*/OR (95%CI)	*P-*value
MBE						
Eosinophils (standardized, per 1-SD increase)*	0.152 (0.066, 0.351)	0.00001	0.160 (0.069, 0.370)	0.00002	0.165 (0.055, 0.498)	0.00137
Eosinophils (dichotomous)**	0.222 (0.136, 0.360)	<0.00001	0.230 (0.141, 0.375)	<0.00001	0.168 (0.086, 0.328)	<0.00001
Poor outcome (mRS score >2)						
Eosinophils (standardized, per 1-SD increase)*	0.574 (0.444, 0.742)	0.00002	0.587 (0.456, 0.755)	0.00003	0.620 (0.475, 0.810)	0.00045
Eosinophils (dichotomous)**	0.215 (0.137, 0.337)	<0.00001	0.218 (0.136, 0.348)	<0.00001	0.179 (0.096, 0.333)	<0.00001
mRS						
Eosinophils (standardized, per 1-SD increase)*	−0.462 (−0.641, −0.283)	<0.00001	−0.426 (−0.594, −0.258)	<0.00001	−0.304 (−0.480, −0.128)	0.00081
Eosinophils (dichotomous)**	−1.437 (−1.783, −1.092)	<0.00001	−1.270 (−1.597, −0.942)	<0.00001	−1.073 (−1.421, −0.726)	<0.00001

Non-adjusted model: we did not adjust other covariates. Model 1: we adjusted age and sex. Model 2: we adjusted variables which were significantly associated with outcomes of interest (*p* < 0.10) or changed the estimates of eosinophils on outcomes of interest by more than 10%. CI, confidence interval; MBE, malignant brain edema; mRS, modified Rankin Scale. *Standardized eosinophils^*^: Z-score transformed using the formula Z = (X - 0.026) / 0.049, where X is the raw eosinophil count, 0.026 is the mean, and 0.049 is the standard deviation of the original distribution. OR indicates the risk change per 1 standard deviation increase in eosinophil count. ^**^Dichotomous eosinophils: Categorized at the median value of 0.01* 10^9/L (low vs. high).

Subgroup analyses further explored the above associations between eosinophils and MBE. As shown in Appendix Table VII, interaction tests showed no significant differences for sex, diabetes, hyperlipidemia, smoking, alcohol consumption, history of stroke, baseline NIHSS, or prior intravenous thrombolysis (all *P* for interaction > 0.05). Three notable significant interaction effects were identified. First, age exerted a modifying effect (*P* for interaction = 0.0149): the inverse association between eosinophils and MBE was much stronger in patients aged ≥ 68 years than in younger counterparts. Second, a prominent interaction was observed for hypertension (*P* for interaction = 0.0019), indicating that the protective association was more evident in patients with hypertension. Third, atrial fibrillation also modified the relationship (*P* for interaction = 0.0334): the inverse association between eosinophils and MBE was statistically significant in patients without atrial fibrillation, while the effect was attenuated in those with atrial fibrillation. Additionally, the independent association between eosinophils and MBE was more prominent in patients without coronary heart disease (*p* = 0.0378).

### Mediation analysis for functional outcome

Eosinophils were independently associated with poor 3-month functional outcome (mRS > 2). For the standardized continuous variable, the adjusted OR was 0.620 (95% CI: 0.475–0.810; *p* = 0.00045); for the dichotomized variable, the OR was 0.179 (95% CI: 0.096–0.333; *p* < 0.00001) (Table 2). Because BBB disruption is central to MBE pathogenesis and eosinophils have been reported to exert potential neuroprotective effects in preclinical models, we constructed a mediation model to explore the potential statistical intermediary role of MBE.

Mediation analysis suggested that MBE statistically accounted for a small proportion of the association between eosinophils and poor outcome (Figure 1A). The proportion of the total association statistically mediated by MBE was 12.05% (95% CI: 2.92–26%; *p* < 0.001). After accounting for the statistical mediating effect of MBE, the direct effect of eosinophils on poor outcome remained statistically significant (*p* < 0.001). The indirect effect (indirect effect = −0.2109; 95% CI: −0.4016 to −0.0500; *p* < 0.001) indicates that higher eosinophil levels are associated with lower risk of poor outcome in part through lower incidence of MBE. The mediation effect on mRS scores was also quantified (Figure 1B).

**Figure 1:**
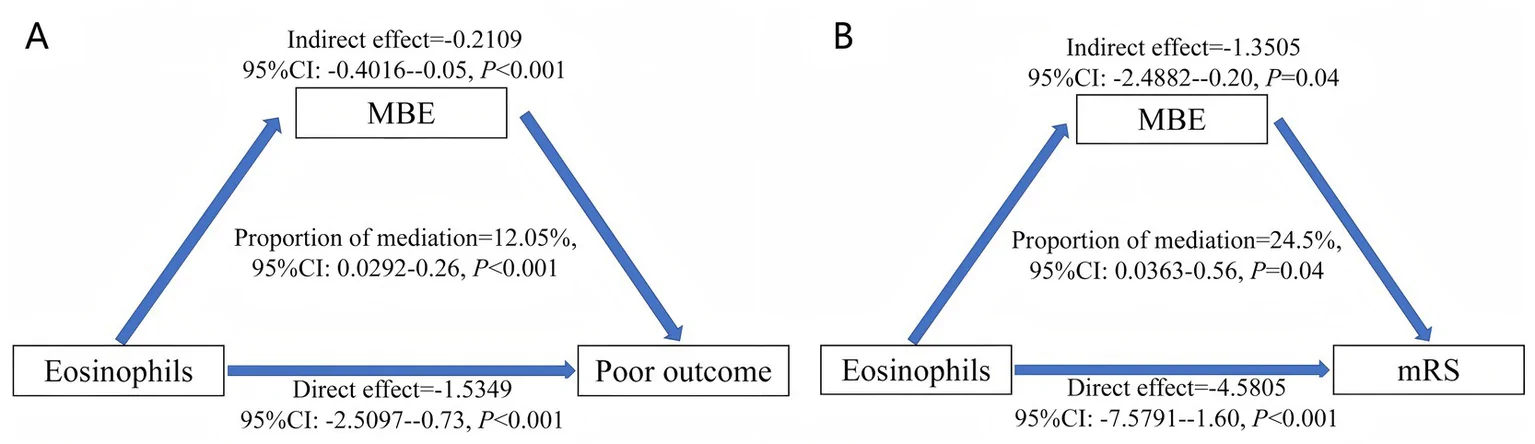
Schematic diagram of statistical mediation analyses for functional outcome. **(A)** Binary poor outcome (modified Rankin Scale score 3-6); **(B)** ordinal modified Rankin Scale score. Eosinophils were entered as predictors and malignant brain edema as the statistical mediator. All mediation analyses were exploratory and do not establish biological causation.

## Discussion

Endovascular MT has become the standard reperfusion therapy for patients with AIS caused by large vessel occlusion. MBE is a life-threatening post-procedural complication closely associated with increased 3-month mortality and poor outcome (3, 11, 15, 16). In this single-center retrospective study of 442 AIS patients receiving MT, we found that lower admission eosinophil counts were independently associated with a higher risk of MBE. Consistent with previous studies (2, 10), eosinophil levels were also inversely associated with 3-month functional outcome. A key finding from mediation analysis was that MBE statistically accounted for a small proportion of the association between eosinophils and poor outcome (12.05%; 95% CI: 2.92–26%). The small mediated proportion and broad confidence interval indicate that this statistical pathway represents a minor intermediate association. Our analysis quantifies this statistical pathway but does not establish biological causation.

The wide confidence interval of the mediated proportion reflects a well-recognized statistical phenomenon: confidence intervals for proportion mediated are typically wide, particularly when the total effect is modest or the direct effect is close to the null. Accordingly, the point estimate of the mediated proportion should be interpreted with caution, as its magnitude cannot be precisely quantified in this sample. The admission eosinophil count is an inexpensive, widely available laboratory marker, yet its utility for routine MBE risk stratification remains preliminary and requires external multicenter validation before clinical application; no predictive models or diagnostic performance metrics were generated in this study to confirm its incremental value over established stroke scoring systems.

The potential biological mechanisms underlying the relationship between eosinophils and MBE remain speculative. Preclinical studies have indicated that eosinophils can secrete anti-inflammatory cytokines (IL-4, IL-13) and VEGF, which may help maintain BBB integrity and regulate neuroinflammation after cerebral ischemia–reperfusion injury (7–9). Notably, VEGF and related inflammatory mediators are also produced by macrophages and platelets, and post-stroke MBE is a complex pathological process driven by multiple immune cell subsets, inflammatory cascades and oxidative stress. Accordingly, the neuroprotective hypotheses proposed herein are based only on statistical associations and published literature, rather than definitive causal conclusions. Building on our prior work demonstrating that eosinophils affect stroke outcomes through pathways including stroke-associated pneumonia and hemorrhagic transformation (2, 10), the current findings further suggest that eosinophils may affect post-MT functional outcome through multiple pathways, with MBE representing only a minor statistical mediator. This is supported by the observation that the association between eosinophils and poor outcome remained significant after adjustment for MBE (Appendix Table VIII), indicating that additional pathways beyond MBE account for a considerable portion of the total association.

Subgroup interaction analyses further identified heterogeneous associations across clinical characteristics. It should be emphasized that all subgroup analyses are exploratory and were not adjusted for multiple comparisons; the observed interaction effects may be subject to type I error inflation. Significant modifying effects were observed for age, hypertension and atrial fibrillation. Advanced age and chronic hypertension lead to chronic cerebral microvascular damage and inherent BBB fragility, which may increase susceptibility to vasogenic edema and amplify the potential protective role of eosinophils. By contrast, cardioembolic stroke related to atrial fibrillation has distinct thrombo-inflammatory features, which may weaken this association (17, 18). The extremely wide confidence interval in the elderly subgroup indicates limited statistical precision. We also detected a significant interaction for coronary heart disease, but this finding was not further interpreted due to the small sample size and unstable effect estimates in the corresponding subgroup. These subgroup results only generate testable hypotheses and cannot serve as validated criteria for individualized MBE risk stratification at present.

Several critical limitations should be considered when interpreting our results. First and most importantly, all analyses reported herein represent statistical associations rather than causal relationships. Even with a clear temporal sequence, mediation analysis cannot confirm directional biological pathways, and the observed correlations lack mechanistic validation from cellular or animal studies. Accordingly, our findings are hypothesis-generating rather than confirmatory of causation; the term “mediator” is used throughout in its statistical sense only. Second, baseline infarct core volume and final infarct volume were not systematically quantified in this clinical cohort. These variables are among the strongest independent predictors of MBE and may also influence circulating eosinophil counts via stress-mediated immune modulation. We included ASPECTS in all multivariable models—a validated semi-quantitative proxy that correlates strongly with infarct extent—which partially mitigates this confounding. Critically, the association between eosinophils and MBE remained significant after adjustment for ASPECTS. If residual confounding from infarct volume were the sole driver of our findings, the effect would be expected to be substantially attenuated or abolished by adjusting for ASPECTS. The persistence of a significant association suggests that the eosinophil-MBE association is not entirely explained by infarct size. Nevertheless, we cannot exclude residual confounding from unmeasured volumetric data and collateral status, and this represents a major limitation. Future prospective studies with standardized volumetric imaging are needed to confirm our findings. Third, peripheral eosinophils were tested only at admission without serial dynamic monitoring. Eosinophils exhibit circadian rhythms, and variation in onset-to-admission time may introduce measurement noise. However, this variation is non-systematic and would be expected to dilute the observed association rather than produce a spurious positive result. The fact that we still detected a significant association suggests that the underlying signal is robust. Circulating eosinophil levels also do not directly reflect leukocyte infiltration within cerebral ischemic tissue, creating a critical translational gap between peripheral laboratory values and intracranial inflammatory pathology (19, 20). Future studies with standardized or multiple sampling times would be valuable. Fourth, although the 5-day window for MBE assessment was based on established criteria and the typical peak of malignant edema occurs at 2–5 days, we acknowledge that a small number of patients with delayed MBE may have been missed. This would result in non-differential misclassification, which typically biases effect estimates toward the null. Therefore, our findings, if anything, are conservative and unlikely to be false-positive. Fifth, this is a single-center study, and the sample exclusively consists of Chinese patients treated with MT for anterior circulation large-vessel occlusion. This limits generalizability to other ethnic populations, patients treated with intravenous thrombolysis alone, patients with posterior circulation stroke, or those with minor vessel occlusion. Additionally, the exclusion of patients with missing data or posterior circulation infarction may have introduced selection bias. Large multicenter prospective cohorts with serial inflammatory measurements and translational preclinical research are necessary to replicate and elaborate upon our preliminary correlational findings.

## Conclusion

In patients with acute ischemic stroke undergoing MT, lower admission eosinophil counts were independently associated with a higher risk of MBE and poorer 3-month functional outcome. Mediation analysis suggested that MBE statistically accounted for a small proportion of the eosinophil-outcome association. All findings presented herein represent statistical associations rather than causal inferences. Significant interaction effects were observed across age, hypertension, and atrial fibrillation in subgroup analyses, and all subgroup findings are exploratory and hypothesis-generating only. As an easily accessible peripheral laboratory marker, the admission eosinophil count may provide preliminary auxiliary reference for MBE risk stratification, pending external multicenter validation and further predictive model development.

## Data Availability

The raw data supporting the conclusions of this article will be made available by the authors, without undue reservation.

## Footnotes

1
http://www.empowerstats.com
