# Supplementary material for: Eosinophils, malignant brain edema, and functional outcome after mechanical thrombectomy: a mediation analysis
Source: Front Neurol. 2026 Oct 7;17:1939369. doi: 10.3389/fneur.2026.1939369 (PMC13683062; doi:10.3389/fneur.2026.1939369)
Supplement: Supplementary file 1 [file Supplementary_file_1.doc]

**Appendix A**

Eosinophils, malignant brain edema, and functional outcome after mechanical thrombectomy: a mediation analysis

**Table I.** Demographic and clinical characteristics of included and excluded patients

| Characteristics | Patients included | Patients excluded | *P* Value |
| --- | --- | --- | --- |
| No. of patients | 442 | 217 |  |
| Age, y; median (IQR) | 68.00 (58.00-75.00) | 67.50 (54.00-74.00) | 0.253 |
| Female, n (%) | 194 (43.89%) | 57 (30.00%) | 0.001 |
| Atrial fibrillation, n (%) | 201 (45.48%) | 50 (26.74%) | <0.001 |
| Hypertension, n (%) | 300 (67.87%) | 132 (70.59%) | 0.502 |
| Diabetes, n (%) | 86 (19.46%) | 36 (19.25%) | 0.952 |
| Hyperlipidemia, n (%) | 128 (28.96%) | 43 (25.29%) | 0.365 |
| History of stroke, n (%) | 64 (14.48%) | 36 (19.25%) | 0.135 |
| Coronary heart disease, n (%) | 72 (16.29%) | 25 (13.37%) | 0.354 |
| Smoking, n (%) | 139 (31.45%) | 73 (39.04%) | 0.066 |
| Drinking, n (%) | 103 (23.30%) | 55 (29.41%) | 0.106 |
| Baseline NIHSS, median (IQR) | 17.00 (13.00-20.00) | 16.00 (11.00-22.00) | 0.023 |
| IVT, n (%) | 139 (31.45%) | 51 (27.27%) | 0.209 |
| Stroke etiology, n (%) |  |  | <0.001 |
| LAA | 192 (43.44%) | 107 (61.49%) |  |
| Cardioembolic | 223 (50.45%) | 49 (28.16%) |  |
| Others | 27 (6.11%) | 18 (10.34%) |  |

IQR, interquartile range; IVT, intravenous thrombolysis; LAA, large-artery atherosclerosis; mRS, modified Rankin Scale; NIHSS, National Institutes of Health Stroke Scale.

**Table II.** The collinearity screening of baseline characteristics

| Characteristics | Variance inflation factor |
| --- | --- |
| Age | 1.6 |
| Female | 1.7 |
| Atrial fibrillation | 2 |
| Hypertension | 1.3 |
| Diabetes | 1.1 |
| Hyperlipidemia | 1.1 |
| History of stroke | 1.1 |
| Coronary heart disease | 1.1 |
| Smoking | 1.9 |
| Drinking | 1.7 |
| Baseline NIHSS | 1.1 |
| ASPECTS | 1.1 |
| IVT | 1 |
| Stroke etiology | 1.8 |
| OPT | 1.1 |
| Number of passes | 1.2 |
| mTICI score 2b or 3 | 1.1 |
| Eosinophils (standardized)* | 1.4 |
| Eosinophils (dichotomous)** | 1.4 |

ASPECTS; Alberta Stroke Program Early CT Score; IVT, intravenous thrombolysis; mTICI, modified Thrombolysis in Cerebral Infarction; NIHSS, National Institutes of Health Stroke Scale; OPT, onset-to-puncture time.

We think collinearity exists and eliminate these covariables in the final models if their variance inflation factors are greater than or equal to 5.

*Standardized eosinophils*: Z-score transformed using the formula Z = (X - 0.026) / 0.049, where X is the raw eosinophil count, 0.026 is the mean, and 0.049 is the standard deviation of the original distribution. OR indicates the risk change per 1 standard deviation increase in eosinophil count.

**Dichotomous eosinophils: Categorized at the median value of 0.01* 10^9/L (low vs. high).

**Table III.** Associations of covariates with malignant brain edema

| Covariates | exp(beta) | 95%CI Low | 95%CI Upp | P.value |
| --- | --- | --- | --- | --- |
| Age | 1.0038 | 0.9871 | 1.0208 | 0.6563 |
| Female | 1.6957 | 1.0794 | 2.6638 | 0.0219 |
| Hypertension | 1.2391 | 0.7573 | 2.0276 | 0.3934 |
| Diabetes | 1.0800 | 0.6171 | 1.8904 | 0.7875 |
| Hyperlipidemia | 0.7484 | 0.4473 | 1.2523 | 0.2698 |
| Atrial fibrillation | 1.8299 | 1.1624 | 2.8805 | 0.0090 |
| Coronary heart disease | 0.9100 | 0.4900 | 1.6899 | 0.7651 |
| History of stroke | 1.4576 | 0.8014 | 2.6512 | 0.2170 |
| Smoking | 0.9512 | 0.5851 | 1.5463 | 0.8399 |
| Drinking | 0.7444 | 0.4261 | 1.3006 | 0.2998 |
| IVT | 0.6458 | 0.3870 | 1.0777 | 0.0942 |
| Baseline NIHSS | 1.0882 | 1.0507 | 1.1271 | <0.0001 |
| ASPECTS | 0.6972 | 0.5378 | 0.9040 | 0.0065 |
| Stroke etiology |  |  |  |  |
| LAA | Reference | Reference | Reference | Reference |
| Cardioembolic | 1.5589 | 0.9754 | 2.4916 | 0.0635 |
| Others | 0.5417 | 0.1546 | 1.8977 | 0.3378 |
| OPT | 0.9989 | 0.9975 | 1.0004 | 0.1400 |
| Number of passes | 1.5202 | 1.2568 | 1.8387 | <0.0001 |
| mTICI score 2b or 3 | 0.5333 | 0.2688 | 1.0582 | 0.0722 |

ASPECTS, Alberta Stroke Program Early CT Score; IVT, intravenous thrombolysis; LAA, large-artery atherosclerosis; mTICI, modified Thrombolysis in Cerebral Infarction; NIHSS, National Institutes of Health Stroke Scale; OPT, onset-to-puncture time.

**Table IV. The adjusting roles of potential confounders on the estimates of eosinophils on malignant brain edema**

| +/- covariates | Basic model | Complete model | The selected covariates |
| --- | --- | --- | --- |
| Initial regression coefficient of eosinophils | -0.5807 | -0.4779 |  |
| Age | -0.5628 | -0.4464 |  |
| Female | -0.5333 | -0.4707 |  |
| Hypertension | -0.5964 | -0.4776 |  |
| Diabetes | -0.5753 | -0.4766 |  |
| Hyperlipidemia | -0.5697 | -0.4754 |  |
| Atrial fibrillation | -0.5431 | -0.4721 |  |
| Coronary heart disease | -0.6010 | -0.4454 |  |
| History of stroke | -0.5677 | -0.4810 |  |
| Smoking | -0.5233 | -0.4869 |  |
| Drinking | -0.5559 | -0.4697 |  |
| Baseline NIHSS | -0.4490 * | -0.5475 * | Yes |
| ASPECTS | -0.5461 | -0.4852 |  |
| IVT | -0.5716 | -0.4858 |  |
| Stroke etiology | -0.5869 | -0.4312 |  |
| OPT | -0.5775 | -0.4774 |  |
| Number of passes | -0.5410 | -0.4869 |  |
| mTICI score 2b or 3 | -0.5538 | -0.4915 |  |

The selected covariates: these confounders changed the estimates of eosinophils on malignant brain edema by more than 10% when introduce covariates into the basic model or remove covariates from the complete model.

ASPECTS, Alberta Stroke Program Early CT Score; IVT, intravenous thrombolysis; mTICI, modified Thrombolysis in Cerebral Infarction; NIHSS, National Institutes of Health Stroke Scale; OPT, onset-to-puncture time.

**Table V.** The selected covariates

| Y | X | The selected covariates（Criterion 1） | The selected covariates（Criterion 2） |
| --- | --- | --- | --- |
| MBE | Eosinophils (standardized)* |  | Female, Atrial fibrillation, IVT, Baseline NIHSS, ASPECTS, Stroke etiology, OPT, Number of passes, mTICI score 2b or 3 |
| MBE | Eosinophils (dichotomous)** |  | Female, Atrial fibrillation, IVT, Baseline NIHSS, ASPECTS, Stroke etiology, Number of passes, mTICI score 2b or 3 |
| Poor outcome | Eosinophils (standardized)* | Baseline NIHSS, | Age, Female, Hypertension, Diabetes, Atrial fibrillation, Coronary heart disease, History of stroke, Smoking, Drinking, IVT, Baseline NIHSS, ASPECTS, Stroke etiology, Number of passes, mTICI score 2b or 3 |
| Poor outcome | Eosinophils (dichotomous)** |  | Age, Female, Hypertension,  Diabetes, Atrial fibrillation, Coronary heart disease, History of stroke, Smoking, Drinking, IVT, Baseline NIHSS, ASPECTS, Stroke etiology, Stroke etiology, Number of passes, mTICI score 2b or 3 |
| mRS score | Eosinophils (standardized)* | Female, Smoking, Baseline NIHSS, Number of passes | Age, Female, Hypertension,  Diabetes, Atrial fibrillation, Coronary heart disease, History of stroke, Smoking, Drinking, IVT, Baseline NIHSS, ASPECTS, Stroke etiology, OPT, Number of passes, mTICI score 2b or 3 |
| mRS score | Eosinophils (dichotomous)** | Baseline NIHSS | Age, Female, Hypertension,  Diabetes, Atrial fibrillation, Coronary heart disease, History of stroke, Smoking, Drinking, IVT, Baseline NIHSS, ASPECTS, Stroke etiology, OPT, Number of passes, mTICI score 2b or 3 |

Criterion 1: These confounders changed the estimates of eosinophils on the outcomes of interest by more than 10% when introduce covariates into the basic model or remove covariates from the complete model.

Criterion 2: These variables were significantly associated with the outcomes of interest (*P* < 0.10) or changed the estimates of eosinophils on the outcomes of interest by more than 10%.

ASPECTS, Alberta Stroke Program Early CT Score; IVT, intravenous thrombolysis; MBE, malignant brain edema; mRS, modified Rankin Scale; mTICI, modified Thrombolysis in Cerebral Infarction; NIHSS, National Institutes of Health Stroke Scale; OPT, onset-to-puncture time.

*Standardized eosinophils*: Z-score transformed using the formula Z = (X - 0.026) / 0.049, where X is the raw eosinophil count, 0.026 is the mean, and 0.049 is the standard deviation of the original distribution. OR indicates the risk change per 1 standard deviation increase in eosinophil count.

**Dichotomous eosinophils: Categorized at the median value of 0.01* 10^9/L (low vs. high).

**Table VI.** The results of threshold effect analysis

| Outcome: | MBE  OR; (95% CI); *P* Value |
| --- | --- |
| Model I |  |
| A linear model | 0.165 (0.055, 0.498) 0.0014 |
| Model II(two-piecewise linear regression model) |  |
| Inflection point of eosinophils(K) | -0.531 |
| < K | NA |
| > K | 0.165 (0.055, 0.498) 0.0014 |
| *P* for log likelihood ratio test | 1.000 |

Effect: eosinophils; Cause: MBE; Adjusted: variables which were significantly associated with outcomes of interest (*P* < 0.10) or changed the estimates of eosinophils on outcomes of interest by more than 10% (Table II-V in the Appendix A). OR, odds ratio.

Standardized eosinophils*: Z-score transformed using the formula Z = (X - 0.026) / 0.049, where X is the raw eosinophil count, 0.026 is the mean, and 0.049 is the standard deviation of the original distribution. OR indicates the risk change per 1 standard deviation increase in eosinophil count.

**Table VII. Subgroup analyses on the association between eosinophils and MBE among patients with acute ischemic stroke experiencing mechanical thrombectomy**

| Subgroup | N | MBE | | |
| --- | --- | --- | --- | --- |
|  |  | OR (95%CI) | *P* value | *P* for interaction |
| Age, years |  |  |  | 0.0149 |
| ＜68 (Median age) | 219 | 0.33 (0.10, 1.02) | 0.0541 |  |
| ≥68 | 223 | 0.01 (0.00, 0.22) | 0.0047 |  |
| Sex |  |  |  | 0.4280 |
| Male | 248 | 0.11 (0.02, 0.53) | 0.0060 |  |
| Female | 194 | 0.27 (0.06, 1.19) | 0.0835 |  |
| Atrial fibrillation |  |  |  | 0.0334 |
| No | 241 | 0.03 (0.00, 0.30) | 0.0028 |  |
| Yes | 201 | 0.38 (0.12, 1.21) | 0.1005 |  |
| Hypertension |  |  |  | 0.0019 |
| No | 142 | 0.78 (0.31, 1.98) | 0.5986 |  |
| Yes | 300 | 0.03 (0.00, 0.20) | 0.0005 |  |
| Diabetes |  |  |  | 0.7439 |
| No | 356 | 0.15 (0.04, 0.52) | 0.0029 |  |
| Yes | 86 | 0.24 (0.02, 2.59) | 0.2396 |  |
| Hyperlipidemia |  |  |  | 0.8641 |
| No | 314 | 0.15 (0.03, 0.70) | 0.0156 |  |
| Yes | 128 | 0.18 (0.04, 0.89) | 0.0355 |  |
| Coronary heart disease |  |  |  | 0.0378 |
| No | 370 | 0.21 (0.07, 0.62) | 0.0047 |  |
| Yes | 72 | 0.00 (0.00, 2.07) | 0.0663 |  |
| History of stroke |  |  |  | 0.3599 |
| No | 378 | 0.20 (0.06, 0.60) | 0.0044 |  |
| Yes | 64 | 0.03 (0.00, 2.19) | 0.1097 |  |
| Smoking |  |  |  | 0.7575 |
| No | 303 | 0.14 (0.03, 0.63) | 0.0103 |  |
| Yes | 139 | 0.20 (0.04, 0.96) | 0.0447 |  |
| Drinking |  |  |  | 0.7811 |
| No | 339 | 0.18 (0.05, 0.62) | 0.0068 |  |
| Yes | 103 | 0.13 (0.01, 1.27) | 0.0787 |  |
| Baseline NIHSS |  |  |  | 0.5180 |
| ＜17 (Median NIHSS) | 214 | 0.23 (0.06, 0.93) | 0.0389 |  |
| ≥17 | 228 | 0.11 (0.02, 0.60) | 0.0104 |  |
| IVT |  |  |  | 0.0727 |
| No | 303 | 0.25 (0.08, 0.76) | 0.0147 |  |
| Yes | 139 | 0.01 (0.00, 0.76) | 0.0382 |  |

In the multivariate models, confounding factors were included unless the variable was used as a stratification variable.

IVT, intravenous thrombolysis; N, No. of participants; NIHSS, National Institutes of Health Stroke Scale; OR, odds ratio.

Standardized eosinophils: Z-score transformed using the formula Z = (X - 0.026) / 0.049, where X is the raw eosinophil count, 0.026 is the mean, and 0.049 is the standard deviation of the original distribution. OR indicates the risk change per 1 standard deviation increase in eosinophil count.

**Table VIII.** Association of eosinophils with poor outcome was independent of MBE

| Exposure | Non-adjusted | Model 1 | Model 2 |
| --- | --- | --- | --- |
|  | β/OR; (95%CI); *P* Value | β/OR; (95%CI); *P* Value | β/OR; (95%CI); *P* Value |
| mRS |  |  |  |
| Eosinophils (standardized, per 1-SD increase)* | -0.462 (-0.641, -0.283) <0.00001 | -0.426 (-0.594, -0.258) <0.00001 | -0.237 (-0.405, -0.069) 0.00597 |
| Eosinophils (dichotomous)** |  |  |  |
| Low eosinophil level | 0 | 0 | 0 |
| High eosinophil level | -1.437 (-1.783, -1.092) <0.00001 | -1.270 (-1.597, -0.942) <0.00001 | -0.801 (-1.151, -0.451) 0.00001 |
| Poor outcome (mRS score 3-6) |  |  |  |
| Eosinophils (standardized, per 1-SD increase)* | 0.574 (0.444, 0.742) 0.00002 | 0.587 (0.456, 0.755) 0.00003 | 0.651 (0.501, 0.846) 0.00130 |
| Eosinophils (dichotomous)** |  |  |  |
| Low eosinophil level | 1.0 | 1.0 | 1.0 |
| High eosinophil level | 0.215 (0.137, 0.337) <0.00001 | 0.218 (0.136, 0.348) <0.00001 | 0.221 (0.116, 0.420) <0.00001 |

Non-adjusted model: we did not adjust other covariates.

Model 1: we adjusted age and sex.

Model 2: we adjusted variables which were significantly associated with outcomes of interest (*P* < 0.10) or changed the estimates of eosinophils on outcomes of interest by more than 10%.

MBE, malignant brain edema; mRS, modified Rankin Scale; OR, odds ratio.
*Standardized eosinophils*: Z-score transformed using the formula Z = (X - 0.026) / 0.049, where X is the raw eosinophil count, 0.026 is the mean, and 0.049 is the standard deviation of the original distribution. OR indicates the risk change per 1 standard deviation increase in eosinophil count.

**Dichotomous eosinophils: Categorized at the median value of 0.01* 10^9/L (low vs. high).

**Figure I.** Flow chart of patient cohort

AIS: Acute ischemic stroke.
